# Patients' experiences of remote communication after pacemaker implant: The *NORDLAND* study

**DOI:** 10.1371/journal.pone.0218521

**Published:** 2019-06-20

**Authors:** Daniel Catalan-Matamoros, Antonio Lopez-Villegas, Knut Tore-Lappegard, Remedios Lopez-Liria

**Affiliations:** 1 Health Sciences CTS-451 Research Group, University of Almeria, Almeria, Spain; 2 Department of Journalism and Communication, University Carlos III of Madrid, Madrid, Spain; 3 Division of Medicine, Nordland Hospital, Bodø, Norway; 4 Social Involvement of Critical and Emergency Medicine, CTS-609 Research Group, Hospital de Poniente, Almeria, Spain; 5 Institute of Clinical Medicine, Faculty of Health Sciences, University of Tromsø, Tromsø, Norway; 6 Nursing, Physiotherapy and Medicine Department, Faculty of Health Sciences, University of Almeria, Almeria, Spain; Karolinska Institutet, SWEDEN

## Abstract

**Background:**

The concept of ‘patient experience’ has become central to how to improve healthcare. Remote communication with patients is today a frequent practice in healthcare services, showing similar outcomes to standard outpatient care while enabling cost reduction in both formal and informal care. The purpose of this study was to analyse the experiences of people with telemonitoring pacemakers.

**Methods:**

Patients were randomly allocated to either the telemonitoring or hospital monitoring follow-ups. Using the ‘Generic Short Patient Experiences Questionnaire’ (GS-PEQ), as well as an *ad-hoc* survey from the ‘telehealth patient satisfaction survey’ and ‘costs survey’, patients’ experiences were measured six months after the pacemaker implant in a cohort of 50 consecutive patients. The mean age was 74.8 (± 11.75) years and 26 (52%) patients were male of which 1 was lost in follow-up. Finally, 24 patients were followed up with standard hospital monitoring, while 25 used the telemonitoring system. Differences in baseline characteristics between groups were not found.

**Results:**

Findings showed overall positive and similar experiences in patients living with telemonitoring and hospital monitoring pacemakers. Significant differences were found in GS-PEQ concerning how telemonitoring patients received less information about their diagnosis/afflictions (p = 0.046). We did not find significant differences in other items such as ‘confidence in the clinicians’ professional skills’, ‘treatment perception adapted to their situation’, ‘involvement in decisions regarding the treatment’, ‘perception of hospital organisation’, ‘waiting before admission’, ‘satisfaction of help and treatment received’, ‘benefit received’, and ‘incorrect treatment’.

**Conclusions:**

The remote communication of pacemakers was met with positive levels of patients’ experiences similarly to patients in the hospital monitoring follow-up. However, telemonitoring patients received less information. Thus, improving the quality and timing of information is required in telemonitoring patients in the planning and organisation of future remote communication healthcare services for people living with a pacemaker implant.

## 1. Introduction

Cardiovascular diseases are one of the most frequent reasons of disease-associated mortality [1]. In Norway, cardiovascular diseases are one of the main cause-of-death disease groups, with myocardial infarction being one of the five most important specific causes of death [2]. Worldwide around 3 million people have a pacemaker and around 600,000 pacemakers are implanted every year [3]. According to clinical guidelines, patients with an implanted PM need to be followed-up every 3–12 months [4, 5]. Every consultation frequently involves an assessment of the device’s function, cardiac events, and the patient’s clinical status and, if needed, the pacemaker is reprogrammed or pharmacotherapy is changed [4, 6]. In our current ageing population, there are increasing indications with respect to persons carrying an implant. The follow-up is today a substantial load for national health services [7] as well as for patients and their relatives [8, 9]. In fact, it has been shown that people with cardiovascular conditions demand a large amount of follow-ups and hospital admissions in Norway [10]. In this regard, remote communication in cardiology could mitigate the increasing workload of follow-ups of pacemakers [11].

In the last Norwegian Coordination Reform in the healthcare sector [12], remote communication and the use of telehealth strategies were emphasised. Remote communication between healthcare providers and patients is considered today a tool that decreases outpatient consultations and costs [13]. In fact, long-distance communication with implanted devices is now a reality. Recent studies have shown that using telemonitoring in patients with pacemakers can result in similar clinical outcomes to standard outpatient care while allowing more flexible services organisation and greater cost reduction in both formal and informal care [9, 14, 15]. Moreover, remote monitoring (RM) or telemonitoring (TM) systems have potential advantages such as early detection of cardiovascular events and early response to technical problems in the device or alterations in the patient’s clinical condition [16, 17]. TM could represent a possible solution in helping to reduce the number of consultations and travels to hospital, thereby optimising healthcare resources [15].

Pacemakers are devices that help to monitor abnormal heart rhythms and regulate heartbeats being placed in the chest [1,18]. A pacemaker implant is a very important life event, so these patients may have different and particular experiences of living with such a device [1]. In this regard, it is important to analyse their experiences in order to develop high-quality healthcare services [19]. Indeed, the experience of patients is more and more being considered clinically important and the concept of ‘patient experience’ has become relevant and central when it comes to increase the quality of healthcare services [20]. In telehealth, both the technology and users’ experiences are key determinants of the acceptance and accomplishment of these implementations [21].

Although many economic evaluations and outcome studies have been conducted, few have inquired as to how individuals experience living with remote monitoring pacemakers. We found one study analysing patients’ experiences with standard on-clinic monitored pacemakers [22]. A previous literature review [23] found three studies comparing the experiences of people with pacemakers and implantable cardiac defibrillators (ICD) [24–26]. In the field of telemonitoring cardiac devices, one study analysed diverse aspects of remote monitored ICDs, such as ease of use of the system, acceptance, and satisfaction of patients and clinicians [21]. A recent study [20] enrolled 14 patients with an insertable cardiac monitor (ICM) so as to explore patients’ experiences, and claimed the need for studies on other cardiac telemonitoring technologies. We have not found previous studies specifically on patients’ experiences with remote monitoring pacemakers. Therefore, the aim of this study was to explore the experiences of people living with telemonitoring pacemakers. The rationale was to produce pertinent and translatable knowledge for future opportunities in healthcare contexts of these patients and to direct future research.

## 2. Materials and methods

This paper is part of a larger project in Norway, the NORDLAND study (2014–2017), wherein a team has collaborated which includes chronic heart patients with a pacemaker, their relatives, cardiologists, nurses, psychologists, and health communication experts. This study is based on a randomised, non-masked observational design where participants were assigned to either follow-up consultations in the hospital or follow-up by remote communication technologies. Participants were recruited in Nordland Hospital, Bodø, Norway. This hospital with a pacemaker centre covers 170,000 inhabitants and conducts around 80–90 pacemaker implants per year [27].

The following protocol has been described in detail previously [27]. Every patient who was scheduled for a pacemaker implant between August 2014 and October 2015 and met all of the inclusion criteria: aged 18 years or older, capacity to provide informed consent and to manoeuvre the home monitor system, and life expectancy >1 year, as well as none of the exclusion criteria: scheduled for an implantable cardioverter-defibrillator (ICD) or cardiac resynchronisation therapy (CRT) and involvement in other studies at the same time, was invited to participate. A total of 76 patients were screened and 50 patients were included and randomised to either telemonitoring (TM, n = 25) or hospital monitoring (HM, n = 25), before being implanted with the pacemaker. The randomisation process was performed as follows: a person unrelated to the study prepared a total of 50 sealed envelopes, 25 of which included a note reading "tele-monitoring" and 25 a note reading "hospital monitoring". The envelopes were thoroughly mixed and numbered from 1 to 50. When a patient accepted the invitation to participate in the trial and signed the informed consent he or she received a consecutive number and was allocated to follow-up in accordance with the specification included in the corresponding envelope. Thus, the investigators had no knowledge of or influence on the randomisation result prior to inclusion.

Depending on their diagnosis, patients were implanted with either a single (VVIR) or a dual chamber (DDDR) pacemaker. For further characteristics and descriptions of the devices that were received by each group of participants please see the previously published article [27].

Data collection was performed 6 months after surgery, by a phone call from one of the research team members. In total, 24 HM patients and 25 TM patients participated, with each participant answering 20 questions. To assess the experiences of the users with PM, an *ad-hoc* questionnaire was created by merging validated questionnaires assessing users’ experiences. The items included comprised the full version of the Generic Short Patient Experiences Questionnaire (GS-PEQ), adding some questions from the telehealth patient satisfaction survey and a costs survey. The Generic Short Patient Experiences Questionnaire (GS-PEQ) is a short set of questions on user experiences with specialist healthcare that covers certain relevant topics. It was created and has been validated in Norway [28]. The questionnaire includes 10 questions (see questions 1–10 in the S1 File) about the following topics: outcome (2), clinician services (2), user involvement (2), incorrect treatment (1), information (1), organisation (1), and accessibility (1). Additionally, to evaluate other aspects of the telehealth experience by patients who have been implanted with home-monitored pacemakers, we used an adapted version of the telehealth patient satisfaction survey [29] and the costs survey [30]. These surveys are composed of closed- and open-ended questions exploring the patients’ experiences with the home monitoring technology, as well as some specific data on costs that they might have with regard to the pacemaker monitoring (see questions 11–20 in the S1 File).

The protocol was approved by the Regional Ethics Committee–REK Nord (Tromsø, Norway), with the committee´s reference number being as follows: 2014/383/REK Nord. The study was developed in accordance with the precepts of the Declaration of Helsinki. All patients signed the corresponding informed consent prior to their enrolment and appropriate measures were taken to ensure data privacy.

**Fig 1:**
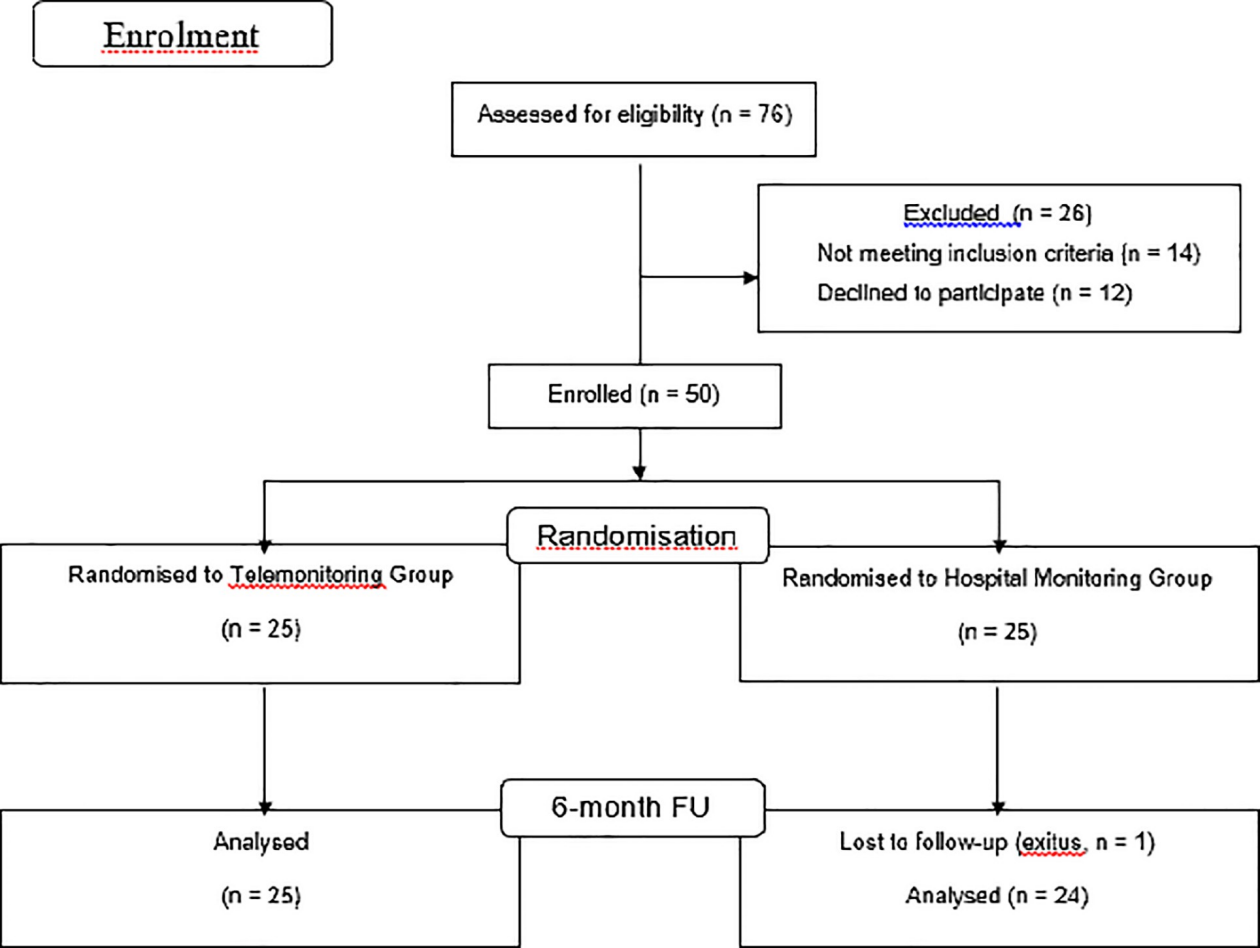
Flow (CONSORT) diagram of the study.

With regard to statistical analyses, following a similar approach of a previous study [9], first patient baseline characteristics and potential differences between groups were compared using a difference in means test for continuous variables and a difference in proportions test (binomial method) or chi-square test (replaced by the Fisher exact test for cells with n<5 cases) for qualitative variables. Secondly, results from the questionnaire were presented on a single question basis with comparison between the two groups, telemonitoring and hospital monitoring, using the Mann–Whitney U test for ordinal data and the chi-square test for nominal data. Analyses were carried out with SPSS 24th edition (SPSS Institute, Inc., Chicago, IL, USA) statistical software. Fig 1 shows the flow (CONSORT) diagram of the study. This figure has been previously published [27].

## 3. Results

Baseline characteristics are presented in Table 1. This table has been previously published [27]. There were no major differences between the telemonitoring and the hospital monitoring groups with regard to age, gender, pacing indication, and other clinical characteristics. Differences were only found in those items that are directly influenced by the type of monitoring: ‘transmissions from patient’s home’ and ‘calls/letters sent to the patients’.

**Table 1:** Selected patient baseline characteristics by intervention status.

	All (n = 50)	Groups		P-value
		Telemonitoring	Hospital monitoring	
Age	74.84 (± 11.75)	73.68 (± 14.22)	76.00 (± 8.77)	0.676
Men	26 (52.0)	13 (52.0)	13 (52.0)	1.00
**Pacing indication N *(%)***				
Sick sinus syndrome	24 (48.0)	12 (48.0)	12 (48.0)	0.648
Atrioventricular block	20 (40.0)	11 (44.0)	9 (36.0)	
Chronic AF with bradycardia	6 (12.0)	2 (8.0)	4 (16.0)	
**Disease manifestations N *(%)***				
Syncope	14 (28.0)	8 (32.0)	6 (24.0)	0.812
Dizziness	25 (50.0)	12 (48.0)	13 (52.0)	
Dyspnoea	11 (22.0)	5 (20.0)	6 (24.0)	
**Service derived N *(%)***				
Emergency dept.	3 (6.0)	1 (4.0)	2 (8.0)	0.505
Cardiology ward	14 (28.0)	5 (20.0)	9 (36.0)	
Primary healthcare	4 (8.0)	2 (8.0)	2 (8.0)	
Other hospitals	29 (58.0)	17 (68.0)	12 (48.0)	
**Stimulation N *(%)***				
DDDR	44 (88.0)	23 (92.0)	21 (84.0)	0.334
VVIR	6 (12.0)	2 (8.0)	4 (16.0)	
**Comorbidities N *(%)***				
Dislipidemia	27 (54.0)	13 (52.0)	14 (56.0)	0.500
Obesity (BMI >30)	1 (2.0)	0 (0.0)	1 (4.0)	0.500
Tachyarrhythmia	18 (36.0)	7 (28.0)	11 (44.0)	0.189
Hypertension	32 (64.0)	17 (68.0)	15 (60.0)	0.384
**Other comorbidities N *(%)***				
None	18 (36.0)	11 (44.0)	7 (28.0)	0.388
Others	10 (20.0)	6 (24.0)	4 (16.0)	
Coronary heart diseases	22 (44.0)	8 (32.0)	14 (56.0)	
**Pharmaceutical treatment N *(%)***				
Antiaggregants	18 (36.0)	8 (32.0)	10 (40.0)	0.384
Anticoagulants	25 (50.0)	10 (40.0)	15 (60.0)	0.129
Antiarrhythmics	18 (36.0)	7 (28.0)	11 (44.0)	0.189
Antihypertensives	32 (64.0)	18 (72.0)	14 (56.0)	0.189

n = 50 (Telemonitoring group: 25; Hospital monitoring group: 25). Values are expressed as means or proportions. SD: Standard deviation; 95 CI: 95% confidence interval of means; DDDR: Bicameral pacemaker with two electrodes placed in the atrium and in the ventricle; VVIR: Unicameral pacemaker with an electrode in the ventricle with the ability to modulate frequency of stimulation; BMI: Body mass index. Note: this table has been previously published in a previous article [27].

In relation to the results derived from the questionnaire, overall experience with both types of follow-ups was positive and, as presented in Table 2, there were few differences between the home monitoring and the hospital monitoring groups with respect to the individual questions. Using the Mann–Whitney U test, χ2 test and Fisher exact test, we only found significant *p* values in two questions (Q3 and Q12). Q3 asked whether patients obtained sufficient information about their diagnosis/afflictions, and Q12 asked for the time that it takes patients to attend a cardiology consultation. In general, responses showed positive experiences, and although these were also positive, the lowest scoring was for Q4 and Q6, for both the intervention and control group patients. These questions concern treatment and organisational perceptions by patients (see Table 3 and full data set in the S1 File).

**Table 2:** Follow-up information at 6 months.

	All (n = 49)	Groups		P-value
		Telemonitoring	Hospital monitoring	
	**Number of transmissions from hospital N *(%)***			
0	0 (0.0)	0 (0.0)	0 (0.0)	0.26
1	41 (83.7)	21 (84.0)	20 (83.3)	
2	6 (12.2)	2 (8.0)	4 (16.7)	
3	2 (4.1)	2 (8.0)	0 (0.0)	
	**Number of transmissions from patient’s home N *(%)***			
0	29 (59.2)	5 (20.0)	24 (100)	<0.001
3–5	15 (30.6)	15 (60.0)	0 (0.0)	
6–8	5 (10.2)	5 (20.0)	0 (0.0)	
	**Extra transmissions from patient’s home N *(%)***			
0	45 (91.8)	21 (84.0)	24 (100)	0.12
1	1 (2.0)	1 (4.0)	0 (0.0)	
3	3 (6.2)	3 (12.0)	0 (0.0)	
	**Cardiovascular events N *(%)***			
None	46 (93.9)	23 (92.0)	23 (95.8)	0.40
PCI	1 (2.0)	1 (4.0)	0 (0.0)	
Angina	1 (2.0)	0 (0.0)	1 (4.2)	
Lead dislodgement x 2	1 (2.0)	1 (2.0)	0 (0.0)	
	**Calls/letters sent to the patients N *(%)***			
0	27 (55.1)	4 (16.0)	23 (95.8)	<0.001
1	21 (42.9)	20 (80.0)	1 (4.2)	
3	1 (2.0)	1 (4.0)	0 (0.0)	
	**Changes of medication N *(%)***			
0	33 (67.3)	17 (68.0)	16 (66.7)	0.11
1	7 (14.3)	5 (20.0)	2 (8.3)	
2	3 (6.1)	1 (4.0)	2 (8.3)	
3	4 (8.2)	0 (0.0)	4 (16.7)	
4	2 (4.1)	2 (8.0)	0 (0.0)	
	**Changes of pacemaker’s reprogramming N *(%)***			
0	34 (69.4)	16 (64.0)	18 (75.0)	0.34
1	13 (26.5)	7 (28.0)	6 (25.0)	
2	2 (4.1)	2 (8.0)	0 (0.0)	
	**Number of hospitalisations (related or not to pacemaker’s implant) N *(%)***			
0	30 (61.2)	14 (56.0)	16 (66.7)	0.55
1	14 (28.6)	7 (28.0)	7 (29.2)	
2	4 (8.2)	3 (12.0)	1 (4.2)	
5	1 (2.0)	1 (2.0)	0 (0.0)	
	**Number of hospitalisation days (related or not to pacemaker’s implant) N *(%)***			
0	30 (61.2)	14 (56.0)	16 (66.7)	0.54
1–5	12 (24.5)	6 (24.0)	6 (25.1)	
6–10	4 (8.1)	2 (8.0)	2 (8.4)	
+10	3 (6.0)	3 (12.0)	0 (0.0)	
	**Reasons for hospitalisation N *(%)***			
None	30 (61.2)	14 (56.0)	16 (66.7)	0.37
Others	6 (12.3)	3 (12.0)	3 (12.5)	
Cancer	1 (2.0)	1 (4.0)	0 (0.0)	
Coronary problems	9 (18.4)	4 (16.0)	5 (20.8)	
Pacemaker dysfunction	3 (6.1)	3 (12.0)	0 (0.0)	

n = 50 (Telemonitoring group: 25; Hospital monitoring group: 24). Values are expressed as means or proportions. SD: Standard deviation; 95 CI: 95% confidence interval of means; **PCI: Percutaneous coronary intervention**. Note: this table has been previously published in a previous article [27].

**Table 3:** Results derived from the Generic Short Patient Experiences Questionnaire (GS-PEQ).

Question	Answering categories*	Telemonitoring group (n = 23)	Hospital monitoring group (n = 23)	p-value
Question 1†	1 = Not at all; 2 = To a small extent; 3 = To a moderate extent; 4 = To a large extent; 5 = To a very large extent	4 (1, 5)	5 (2, 5)	0.214
Question 2†		5 (4, 5)	5 (3, 5)	0.326
Question 3†		4 (2, 5)	5 (1, 5)	*0.046
Question 4†		4 (3, 5)	5 (3, 5)	0.241
Question 5†		3 (1, 5)	3 (1, 5)	0.091
Question 6†		4 (3, 5)	4 (3, 5)	0.712
Question 7†		5 (3, 5)	5 (3, 5)	0.362
Question 8†		1 (1, 3)	1 (1, 2)	0.613
Question 9†		1 (1, 2)	1 (1, 5)	0.492
Question 10†		4 (1, 5)	5 (3, 5)	0.404
Question 11†	Number of kilometres	65 (1, 500)	40 (1, 300)	.183
Question 12†	1 = <1 hour; 2 = 1–2 hours; 3 = 2–3 hours; 4 = 3–4 hours; 5 = >4 hours	>4 (<1, >4)	2–3 (<1, >4)	*.041
Question 13‡	1 = Public transport	8 (34.8)	3 (13.0)	.323
	2 = Own car	0	1 (4.3)	
	3 = Ambulance	10 (43.5)	10 (43.5)	
	4 = Taxi	1 (4.3)	3 (13.0)	
	5 = Other	4 (17.4)	6 (26.0)	
Question 14‡	1 = Working	1 (4.3)	2 (8.7)	.229
	2 = Unemployed	0	0	
	3 = Pensionist	12 (52.2)	8 (34.8)	
	4 = Sick leave	8 (34.8)	13 (56.5)	
	5 = Other	2 (8.7)	0	
Question 15‡	Yes	3 (13.0)	6 (26.1)	.265
	No	20 (87.0)	17 (73.9)	
Question 16‡	1 = Working	2 (50.0)	4 (57.1)	.368
	2 = Unemployed	0	0	
	3 = Pensioner	1 (25.0)	3 (42.9)	
	4 = Sick leave	1 (25.0)	0	
	5 = Other	0	0	
Question 17‡	Yes	17 (73.9)	15 (65.2)	.522
	No	6 (26.1)	8 (34.8)	
Question 18†		36.55 (0–33.25)	22.10 (0–110.75)	.611
Question 19‡	1 = None	19 (82.6)	22 (95.6)	.200
	2 = Once	3 (13.0)	0	
	3 = Twice	0	0	
	4 = More than 2	1 (4.4)	1 (4.4)	
Question 20‡	1 = None	20 (87.0)	22 (95.6)	.351
	2 = Once	2 (8.7)	0	
	3 = Twice	0	0	
	4 = More than 2	1 (4.3)	1 (4.4)	

*For questions 1–10, the following scoring was used: 1, not at all; 2, to a small extent; 3, to some extent; 4, to a large extent; and 5, to a very large extent.

†Data presented as median (min., max.).

‡Data presented as total number (percentage).

## 4. Discussion

The NORDLAND study explored the patients’ experiences with living with telemonitoring pacemakers. To the best of our knowledge, this is the first study to explore patients’ experiences in respect of living with telemonitoring pacemakers. The findings revealed: i) overall positive experiences in patients living with telemonitoring pacemakers; ii) significant differences in the GS-PEQ between both groups concerning telemonitoring patients receiving less information about their diagnosis/afflictions than those ones in hospital monitoring; iii) significant differences in how TM patients take more time to attend a cardiology consultation at hospital than HM patients; and iv) that no significant differences between groups were found in the rest of the items, such as ‘confidence in the clinicians’ professional skills’, ‘treatment perception adapted to their situation’, ‘involvement in decisions regarding the treatment’, ‘perception of hospital organisation’, ‘waiting before admission’, ‘satisfaction of help and treatment received’, ‘benefit received’, and ‘incorrect treatment’.

The overall positive experience found in our study is confirmed and well aligned with previous studies analysing experiences of telehealth devices. In a previous study evaluating telehealth in primary care [31], patients found that this new remote communication with healthcare providers was a flexible, convenient, easy-to-use and acceptable means of their jointly managing their condition with a responsible health professional. Another study [21] found that the ease of use, satisfaction, and acceptance of remote monitoring of implantable defibrillators appeared to be elevated both for patients and for clinicians. However, in spite of these positive results, we need to be cautious, since the implementation of telehealth in regular healthcare practice is difficult [21]. Indeed, 15–20 years ago, it was reported that most telehealth initiatives had become a failure in daily practice, since poor technical feasibility often results in distrust from users and low levels of satisfaction [32, 33]. Nowadays, we can see that technical problems are very much minimised; thus, users’ experiences are more positive, as our findings have revealed.

Besides the overall positive experiences, one relevant outcome found in our study was that telemonitoring patients received less information about their diagnosis/afflictions than those ones in hospital monitoring. This is an important finding concerning the expectations of the information that are normally provided by conversations with the health professionals and the delivery of pamphlets. We believe that due to the reduction of face-to-face consultations, these TM patients had a lesser chance of posing questions or clarifying issues in relation to their diagnosis with the health professionals. Being well informed is a key ingredient in patients’ subjective experiences [34]. A recent study [20] also found that a characteristic part of the diagnostic process in ICM remote monitoring is that patients experience the feeling of ‘not knowing’ or ‘being uninformed’. According to the authors, this might be a result of “no news is good news” in the home monitoring environment. The monitoring systems only inform the health professionals when something goes wrong. According to the authors, this collides with the needs of patients. Frequent contact with clinicians is needed so as to receive information on the health status, especially for patients who are asymptomatic (as this contact is the only way in which to know about their progress). Other previous studies [24, 25] carried out on patients with hospital monitoring cardiac implants also support the recommendations of providing more information such as preceding to implantation. Among the patients, these studies found more fear of death, of device malfunction, greater concerns about not being able to work, and more worry with regard to having sex and driving. These studies recommend that as these patients are mentally ill from depression or anxiety, they suggest that health professionals should be aware of significant symptoms, meeting the patients’ needs. Although we did not find similar studies on telemonitoring patients with pacemakers, we believe that these patients might have higher information needs than hospital monitoring, since their face-to-face or phone contact with healthcare professionals is lower and, therefore, they might have fewer opportunities to obtain information. This may lead us to an Albert Einstein quote: “It has become appallingly obvious that our technology has exceeded our humanity.” In this regard, we agree with Boriani et al. [11] in suggesting that the evolving capabilities of implanted devices to monitor patients’ cardiac status (heart rhythm, fluid overload, right ventricular pressure, oximetry, etc.) may imply a shift from strictly device-centred follow-up to perspectives centred on the patient (and patient–device interactions). A patient-centred approach could provide improvements in healthcare delivery and clinical outcomes in telemonitoring patients.

Finally, we should mention that we found another difference between groups in Q12, which asked for the time that it takes patients to attend a cardiology consultation. However, we believe that this significant difference was a result of chance in spite of the p-value, as all participants were randomly allocated to one of both groups, either telemonitoring or hospital monitoring follow-up.

Despite the relevant results obtained, the study has certain limitations. Firstly, some issues in relation to measuring patient experience include the feedback biases by answering intentionally to the questionnaires in order to accomplish positive outcomes. Some patients are also confused because they consider the questions to be on their experience of their health condition [35]. Secondly, this is an open study wherein clinicians, researchers and patients knew the follow-up category for all participants. Nevertheless, we believe that our study presents some important strong points since the NORDLAND study is a randomised study in a field where it is not frequent to conduct such method design. This ensures a major evidence level, a lesser chance of bias due to random selection of the groups, and it might be repeatable and comparable with other studies [27].

## 5. Conclusions

Our results showed that patients living with telemonitoring pacemakers have overall positive experiences, similar to those with hospital monitoring pacemakers. Areas for improvement should focus on improving the quality and timing of information during the entire therapeutic process. To the best of our knowledge, this is the first publication to show patients’ experiences of living with telemonitoring pacemakers.

Our study also includes some important practical implications. We believe that by achieving a better understanding of the experiential dimensions of these patients we can inform communication practices between healthcare professionals and patients in the follow-up process of a pacemaker implant. In fact, such little research into patient experiences of living with telemonitoring pacemakers could also be a result of the poor educational preparation of health professionals responsible for these patients. Therefore, our findings will support the development of this important research field [36, 37]. Further organisation and management of healthcare services should ensure the delivery of quality and timely information to patients during the entire process, from pre-implantation to the follow-up phases.

## Supporting information

S1 File Supporting information: Variables, coding and anonymous data set. (PDF)
