# Correction: Patients' experiences of remote communication after pacemaker implant: The *NORDLAND* study

**DOI:** 10.1371/journal.pone.0219584

**Published:** 2019-07-05

**Authors:** Daniel Catalan-Matamoros, Antonio Lopez-Villegas, Knut Tore-Lappegard, Remedios Lopez-Liria

The third author’s name is incorrect. The correct name is: Knut-Tore Lappegard. The correct citation is: Catalan-Matamoros D, Lopez-Villegas A, Tore-Lappegard K, Lopez-Liria R (2019) Patients' experiences of remote communication after pacemaker implant: The *NORDLAND* study. PLoS ONE 14(6): e0218521. https://doi.org/10.1371/journal.pone.0218521
