# Correction: Correction: Patients' experiences of remote communication after pacemaker implant: The *NORDLAND* study

**DOI:** 10.1371/journal.pone.0220828

**Published:** 2019-07-31

**Authors:** The PLOS ONE Staff

There is an error in the Correction published on July 5, 2019. The third author’s name is incorrect in the citation. The publisher apologizes for this error.

The correct citation is: Catalan-Matamoros D, Lopez-Villegas A, Lappegard KT, Lopez-Liria R (2019) Patients' experiences of remote communication after pacemaker implant: The *NORDLAND* study. PLoS ONE 14(6): e0218521. https://doi.org/10.1371/journal.pone.0218521.

## References

[1] Catalan-Matamoros D, Lopez-Villegas A, Tore-Lappegard K, Lopez-Liria R (2019) Patients' experiences of remote communication after pacemaker implant: The *NORDLAND* study. PLoS ONE 14(6): e0218521 10.1371/journal.pone.0218521 31220146 PMC6586402

[2] Catalan-Matamoros D, Lopez-Villegas A, Tore-Lappegard K, Lopez-Liria R (2019) Correction: Patients' experiences of remote communication after pacemaker implant: The *NORDLAND* study. PLoS ONE 14(7): e0219584 10.1371/journal.pone.0219584 31276574 PMC6611616

