# Supplementary material for: Patients' experiences of remote communication after pacemaker implant: The NORDLAND study
Source: PLoS One. 2019 Jun 20;14(6):e0218521. doi: 10.1371/journal.pone.0218521 (PMC6586402; doi:10.1371/journal.pone.0218521)
Supplement: S1 File — (PDF) [file pone.0218521.s001.pdf]

NORDLAND STUDY – SUPPORTING INFORMATION

Item	Variable	Coding
Variable 1	Type of follow up	1 = Remote<br>2 = Hospital
Variable 2	Sex	0 = man<br>1 = woman
**The Generic Short Patient Experiences Questionnaire – GS-PEQ (validated by Strømseng Sjetne, 2011) (n=49, n=25 remote, n=24 hospital, n=1 exitus)**		
Question 1	Did the clinicians talk to you in a way that was easy to understand?	1 = Not at all<br>2 = To a small extent<br>3 = To a moderate extent<br>4 = To a large extent<br>5 = To a very large extent
Question 2	Do you have confidence in the clinicians' professional skills?	1 = Not at all<br>2 = To a small extent<br>3 = To a moderate extent<br>4 = To a large extent<br>5 = To a very large extent
Question 3	Did you get sufficient information about your diagnosis/afflictions?	1 = Not at all<br>2 = To a small extent<br>3 = To a moderate extent<br>4 = To a large extent<br>5 = To a very large extent
Question 4	Did you perceive the treatment as adapted to your situation?	1 = Not at all<br>2 = To a small extent<br>3 = To a moderate extent<br>4 = To a large extent<br>5 = To a very large extent
Question 5	Were you involved in decisions regarding your treatment?	1 = Not at all<br>2 = To a small extent<br>3 = To a moderate extent<br>4 = To a large extent<br>5 = To a very large extent
Question 6	Did you perceive the institution's work as well organized?	1 = Not at all<br>2 = To a small extent<br>3 = To a moderate extent<br>4 = To a large extent<br>5 = To a very large extent
Question 7	Did you have to wait before you were admitted for services at the institution?	1 = Not at all<br>2 = To a small extent<br>3 = To a moderate extent<br>4 = To a large extent<br>5 = To a very large extent
Question 8	Overall, was the help and treatment you received at the institution satisfactory?	1 = Not at all<br>2 = To a small extent<br>3 = To a moderate extent<br>4 = To a large extent<br>5 = To a very large extent
Question 9	Overall, what benefit have you had from the care at the institution?	1 = Not at all<br>2 = To a small extent<br>3 = To a moderate extent<br>4 = To a large extent<br>5 = To a very large extent
Question 10	Do you believe that you were in any way given incorrect treatment?	1 = Not at all<br>2 = To a small extent<br>3 = To a moderate extent

		4 = To a large extent<br>5 = To a very large extent
**Survey on telehealth patient experience<br>(adapted version from Abrams & Geier, 2006; Bas-Villalobos, 2010)<br>(n=48, n=24 remote, n=24 hospital)**		
Question 11	How many kilometers is your home from hospital?	
Question 12	How much time does it take you to attend a cardiology consultation?	1 = < 1 hour<br>2 = 1 - 2 hours<br>3 = 2 - 3 hours<br>4 = 3 - 4 hours<br>5 = > 4 hours
Question 13	What type of transport do you use to travel to hospital?	1 = Public transport<br>2 = Own car<br>3 = Ambulance<br>4 = Taxi<br>5 = Other
Question 14	Which is your labour situation now?	1 = Working<br>2 = Unemployed<br>3 = Pensioner<br>4 = Sick leave<br>5 = Other
Question 15	Do you need to be accompanied by any relative or friend to attend the cardiology consultation at hospital?	1 = No<br>2 = Yes
Question 16	Which is the labour situation of your accompanying person?	1 = Working<br>2 = Unemployed<br>3 = Pensioner<br>4 = Sick leave<br>5 = Other
Question 17	Have you or the accompanying person any expenses when travelling to hospital?	1 = No<br>2 = Yes
Question 18	Budget spent	In euros
Question 19	How many times have you phoned to the pacemakers office at hospital?	1 = None<br>2 = 1<br>3 = 2<br>4 = More than 2
Question 20	How many times have you attended the emergency ward for a problem related to your pacemaker in either the hospital or primary healthcare centre?	1 = None<br>2 = 1<br>3 = 2<br>4 = More than 2

Variables<br>Patient code	V1	V2	Q1	Q2	Q3	Q4	Q5	Q6	Q7	Q8	Q9	Q10	Q11	Q12	Q13	Q14	Q15	Q16	Q17	Q18	Q19	Q20
**1**	1	0	4	5	4	4	2	4	4	1	1	4	100	5	3	3	1		2	332,25	1	1
**2**	2	1	5	5	5	5	5	4	4	1	1	5	1	1	3	3	1		2	4,43	1	1
**3**	1	0	5	5	4	5	2	5	5	1	2	5	25	3	3	3	1		1	0	1	1
**4**	1	0	5	5	5	5	4	5	5	1	1	4	1	2	3	1	1		1	0	1	1
**5**	2	1	4	4	4	4	4	4	5	1	1	4	60	4	5	3	1		1	0	1	1
**6**	1	0	5	4	3	3	3	3	3	1	1	3	80	4	5	3	2	1	2	44,3	1	1
**7**	1	0	3	4	2	3	1	3	3	1	1	3	30	3	5	3	2	1	2	11,08	1	1
**8**	2	1	4	5	5	5	4	5	5	1	2	3	73	4	5	4	1		1	0	1	1
**9**	2	1	5	5	5	5	4	4	5	1	1	5	1	1	5	4	1		2	22,15	1	1
**10**	1	0	4	4	4	5	4	4	4	2	1	4	30	5	1	5	1		2	16,61	2	4
**11**	1	0	5	5	4	4	2	4	5	1	2	4	90	5	1	3	1		2	27,69	1	1
**12**	1	0	4	5	5	5	3	5	5	1	1	4										
**13**	1	0	5	5	3	4	2	6	3	1	1	1		5	4	3	1	3	2	27,69	1	1
**14**	2	1	5	4	4	5	1	3	4	1	1	4	70	4	2	3	1		1	0	1	1
**15**	1	0	5	5	4	5	2	4	5	1	2	5	60	5	1	3	1		1	0	1	1
**16**	1	0	5	5	5	5	5	5	5	1	2	5	70	4	3	3	1		2	22,15	1	1
**17**	2	1	5	5	5	4	5	4	5	1	1	4	70	5	3	3	1		1	0	1	1
**18**	1	0	3	4	2	4	1	4	4	1	2	3	55	5	5	3	1		2	55,38	1	1
**19**	2	1	4	5	5	5	6	5	5	1	2	5	130	5	5	4	1		1	0	1	1
**20**	2	1	5	4	4	5	1	4	4	1	3	6	40	3	3	4	1		2	44,3	1	1
**21**	2	1																				
**22**	1	0	5	5	4	4	1	4	4	1	1	4	19	3	3	4	1		2	11,08	1	1
**23**	2	1	5	5	5	5	2	4	5	1	1	4	20	3	4	4	2	1	2	35,44	1	1
**24**	2	1	5	5	5	5	4	4	4	1	1	5	1	2	5	4	1	1	2	22,15	1	1
**25**	2	1	4	5	4	4	4	5	5	1	1	5	78	5	3	4	2	1	1	0	1	4

**26**	2	1	5	5	5	5	5	4	4	1	4	5	100	2	3	4	2		2	33,23	4	1
**27**	2	1	2	5	1	3	1	5	5	1	1	3	300	5	1	4	2	3	2	110,75	1	1
**28**	1	0	4	4	4	4	2	4	5	1	1	4	100	5	1	4	1		2	15,51	1	1
**29**	1	0	4	4	4	4	3	4	4	1	1	5	1	2	5	4	1		1	0	1	1
**30**	2	1	4	5	4	4	4	4	4	1	1	5	8	5	1	3	1		2	55,38	1	1
**31**	2	1	5	5	5	5	1	5	5	1	1	5	3	2	4	4	2	1	2	22,15	1	1
**32**	1	0	4	4	3	4	3	4	5	1	1	5	120	5	3	4	1		2	22,15	1	2
**33**	2	0	5	5	5	5	2	5	5	1	1	5	10	3	3	4	1		2	8,86	1	1
**34**	1	0	5	5	4	5	2	4	4	1	1	5	165	5	3	4	1		2	16,61	1	1
**35**	2	0	5	5	4	5	2	4	4	1	1	5	6	2	3	4	1		1	0	1	1
**36**	1	1	4	5	4	3	3	4	3	3	1	2	4	2	1	4	1		2	33,23	2	2
**37**	2	1	4	3	5	5	3	3	3	1	1	4	60	3	5	3	1	3	2	11,08	1	1
**38**	1	1	5	5	5	5	5	5	5	1	1	5	500	5	1	5	1		1	0	1	1
**39**	2	0	4	5	4	5	2	4	5	1	2	4	200	5	3	3	2	3	2	66,45	1	1
**40**	2	0	5	5	5	5	2	5	5	1	2	5	50	4	3	4	1		2	22,15	1	1
**41**	1	0	1	5	5	5	2	5	5	1	1	5	20	3	3	3	1		2	50	1	1
**42**	2	0	5	4	4	4	4	4	4	2	1	5	8	2	1	1	1		2	5,54	1	1
**43**	1	1	5	5	4	4	4	4	4	1	2	5	80	5	3	3	1		2	77,53	2	1
**44**	2	1	5	4	5	5	4	4	4	1	1	5	3	2	4	3	1		2	22,15	1	1
**45**	2	1	5	5	2	4	1	5	5	1	1	5										
**46**	1	1	4	4	4	4	3	4	5	1	1	4	300	5	1	3	1		2	77,53	4	1
**47**	1	0	3	5	3	5	3	4	5	1	1	1	1	1	3	4	2	4	1	0	1	1
**48**	1	0	4	4	4	5	3	4	5	1	1	5	370	5	1	4	1		2	0	1	1
**49**	2	0	5	5	5	5	2	5	5	1	1	4	5	1	3	1	1		1		1	1
**50**	1	1	5	5	5	5	5	5	5	1	1	4										
